# A novel high-throughput assay to quantify the vaccine-induced inhibition of *Bordetella pertussis* adhesion to airway epithelia

**DOI:** 10.1186/s12866-016-0829-x

**Published:** 2016-09-15

**Authors:** Elisa Zanaboni, Vanessa Arato, Mariagrazia Pizza, Anja Seubert, Rosanna Leuzzi

**Affiliations:** GSK Vaccines, Via Fiorentina 1, 53100 Siena, Italy

**Keywords:** Adhesion assay, Airway epithelium, Fluorescent-labeled bacteria, *B. pertussis*

## Abstract

**Background:**

Pertussis or whooping cough is an acute respiratory illness caused by the Gram-negative pathogen *Bordetella pertussis*. Despite high vaccination coverage whooping cough is currently re-emerging in many developed countries. Although the causes of pertussis resurgence are matter of debate, emerging evidences suggest that acellular vaccines efficiently protect against the hallmark symptoms of pertussis disease but fail to prevent colonization. This presumably impacts on increased risk of bacterial transmission and consequent spread throughout the population. These evidences suggest that improved vaccines may be required for efficient bacterial clearance in the upper respiratory tract. Consequently, there is a need for novel bioassays to evaluate at pre-clinical or clinical level the impact of different vaccines on *B. pertussis* colonization.

**Results:**

We developed a high-throughput bacterial adhesion inhibition (BAI) assay based on human respiratory cell lines and on live bacteria chemically conjugated to a fluorescent dye. Employing A549 cells as model, we evaluated the impact of antibodies elicited by acellular (aP) and whole cell (wP) vaccines on *B. pertussis* adhesion in vitro. Moreover, we settled the method also on polarized Calu-3 cells grown at air-liquid interface (ALI), showing that this assay can be extended to more complex cell models mimicking the airway epithelium.

**Conclusions:**

We proved that this method is a sensitive, rapid and reproducible system to evaluate the anti-adhesive properties of vaccine-induced antibodies and can be employed to assess improved pertussis vaccines.

## Background

Pertussis or whooping cough is an acute respiratory disease caused by *Bordetella pertussis* [1]. Despite high vaccination coverage, pertussis is a re-emerging disease causing mortality in infants worldwide, mainly in developing countries, but also at increasing incidence in many developed countries [2, 3]. While pertussis resurgence is likely caused by multiple factors [4–7], it is becoming increasingly evident that currently licensed acellular pertussis (aP) vaccines are suboptimal in inducing long lasting protection and in preventing colonization. This presumably has an impact on increased risk of transmission and consequent bacterial spread throughout the population. Recent studies in a nonhuman primate model support this hypothesis, demonstrating that aP vaccines efficiently protect against the hallmark symptoms of pertussis disease but fail to prevent colonization [8].

Improvement of current pertussis vaccines or development of novel vaccines should take these findings into consideration [9] and be effective in shortening bacterial colonization besides preventing the disease.

Hurdles for the development of improved vaccines addressing these issues are the lack of pre-clinical tools, such as serological bioassays and adequate animal models, suitable to evaluate bacterial adherence and clearance. The mouse aerosol challenge model, as well as other small animal models, has been useful in dissecting the mechanisms of pathogenesis of *B. pertussis* [10]. However, being *B. pertussis* an obligate human pathogen, these animal models are unable to reproduce the full spectrum of disease in humans. The recently developed baboon model [11] provides an excellent tool to improve our knowledge on *B. pertussis* transmission and to explore the effect of the vaccine-induced immunity on colonization and disease manifestations. However, ethical concerns and high costs limit the use of baboon model for vaccine antigen screening, although it is the model of choice for evaluation of advanced vaccine formulation. Accordingly, new in vitro bioassays mimicking the natural site of infection of *B. pertussis* could represent a valid screening method to test the functionality of vaccine-induced antibodies in inhibiting the bacterial adhesion.

One of the mechanisms to control the bacterial burden is the presence of functional antibodies at the epithelial barrier which could inhibit the attachment of bacteria to the respiratory epithelium and the initiation of colonization. Adhesion assays employing cell models representing the human respiratory tract have been extensively used to study the mechanisms of *B. pertussis* adhesion [12–15]. These studies allowed the identification of the key adhesins involved in the colonization process by the use of mutant strains or by inhibition with antibodies targeting individual virulence factors. However, the adhesion assays employed so far were generally based on bacterial colony counting or microscopic examinations which are time-consuming and inadequate for extensive comparative analysis of the kinetics of bacterial adhesion. To overcome the limitations of conventional adhesion assays and compare the adhesion inhibitory properties of vaccine-induced antibodies, a quantitative assay compatible with high-throughput methodologies is then needed.

We report here the development of a new assay to quantify the adhesion of *B. pertussis* to airway epithelial cells and show the efficiency of this method in evaluating the ability of the antibodies induced by different pertussis vaccine formulations to inhibit bacterial adhesion.

## Results and discussion

### Analysis of fluorescent bacteria

**Fig. 1:**
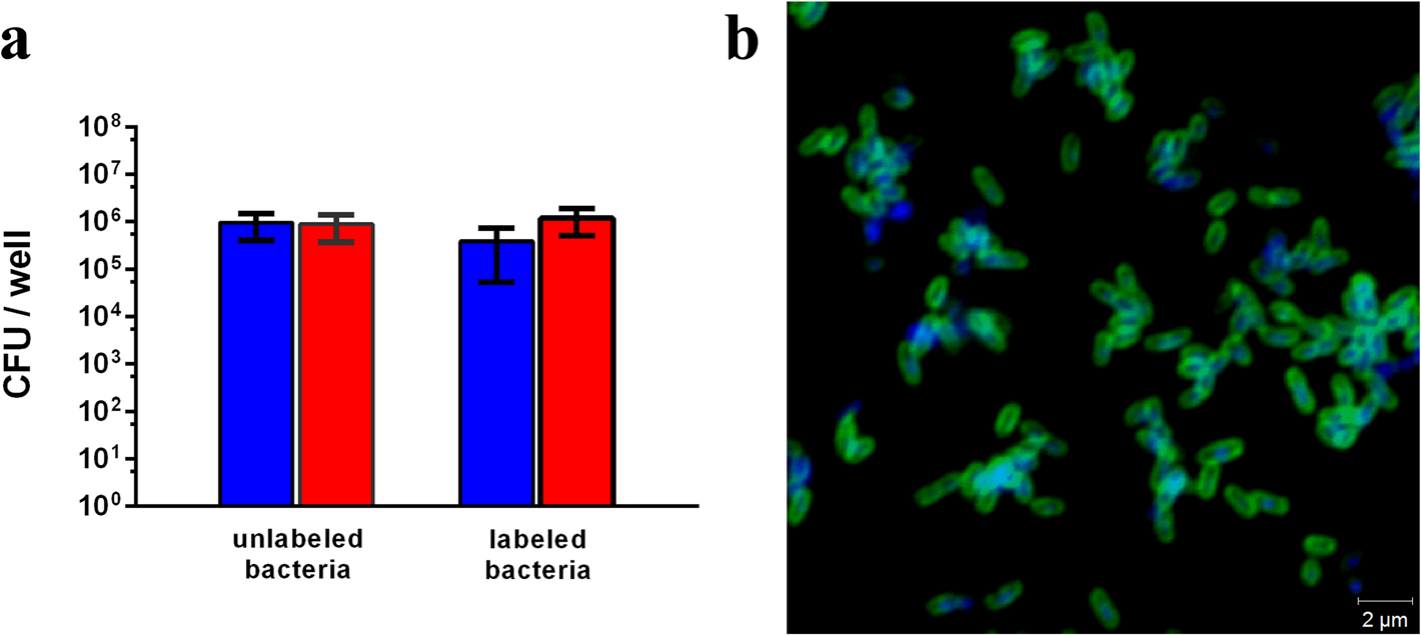
Effect of fluorescent labeling on *B. pertussis* viability. **a** Conjugation of *B. pertussis* Tohama I with Alexa Fluor® 488 was performed as described in Materials and Methods. After washes, bacteria were suspended in PBS. Bacterial suspension was serially diluted and plated on BG agar plates immediately after labeling (*blue*), and 2 h (*red*) after incubation at 37 °C. Results represent mean ± SD from a representative experiment performed in triplicates. **b** Labeled bacteria were fixed (*green*) and stained with DAPI (*blue*) for confocal microscopy analysis

*B. pertussis* bacteria were labeled with a fluorescent dye (Alexa Fluor® 488 Carboxylic Acid, Succinimidyl Ester), which conjugates to the primary amines (R-NH_2_) of amine-containing molecules on bacterial surface. After incubation with the Alexa Fluor® 488, bacteria were washed to remove unbound dye. To assess whether the conjugation affected bacterial viability, bacteria were plated after the conjugation, and the colonies were compared to unconjugated bacteria as control. Colony forming unit (CFU) counting demonstrated that viability of labeled and unlabeled bacteria was comparable and that the survival is maintained after two hours incubation in cell medium (Fig. 1a). Moreover, all bacteria were efficiently labeled as shown by fluorescent microscopy examination (Fig. 1b).

Fluorescent bacteria can be quantified by a multi-plate fluorescent reader, overcoming the limitations of conventional colony counting. To test the sensitivity of the method and to determine the minimum number of bacteria that could be detected by the fluorescence reader, bacteria were serially diluted and each bacterial suspension was measured for fluorescence emission and plated for colony counting.

**Fig. 2:**
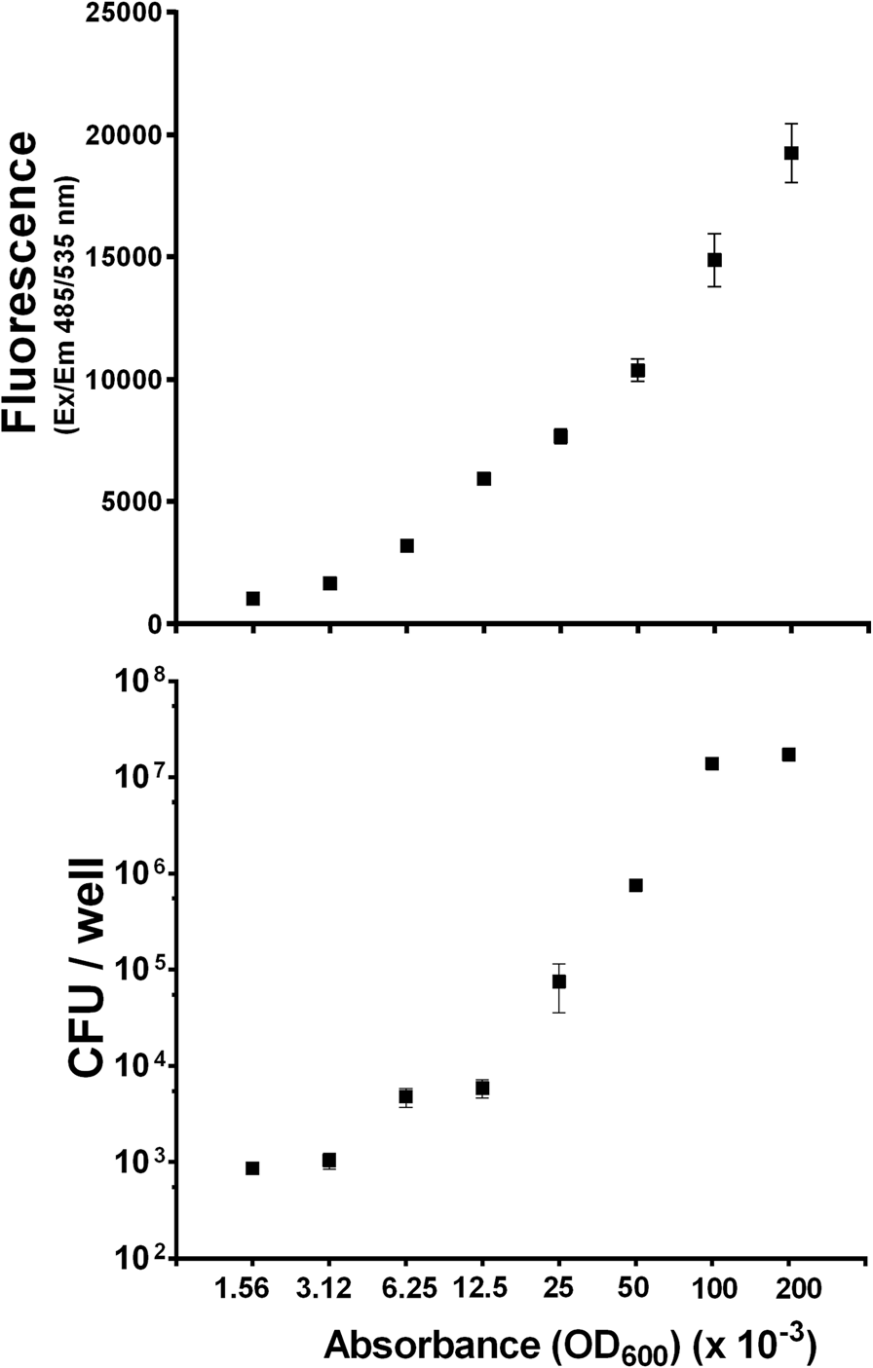
Standard curve showing the relationship between bacteria-associated fluorescence and number of bacteria. Labeling of *B. pertussis* Tohama I with Alexa Fluor® 488 was performed as described in Materials and Methods. Fluorescent bacteria were suspended in PBS to reach OD_600_ 0.2 and then 2-fold serially diluted. For each dilution, 100 μL of bacterial suspension were transferred in triplicates in a 96-well plate and fluorescence was assessed at 485/535 nm by a multi-well fluorescent reader, while 100 μL were serially diluted and plated on BG agar plates in triplicates for CFU counting. Results represent mean ± SD from one out of the two representative experiments performed in triplicates

Figure 2 shows a linear relationship between the number of bacteria and fluorescence and establishes that the minimum number of bacteria that could be detected by the reader is in the range of 10^3^ CFU.

### Development of a high-throughput adhesion assay for *B. pertussis*

**Fig. 3:**
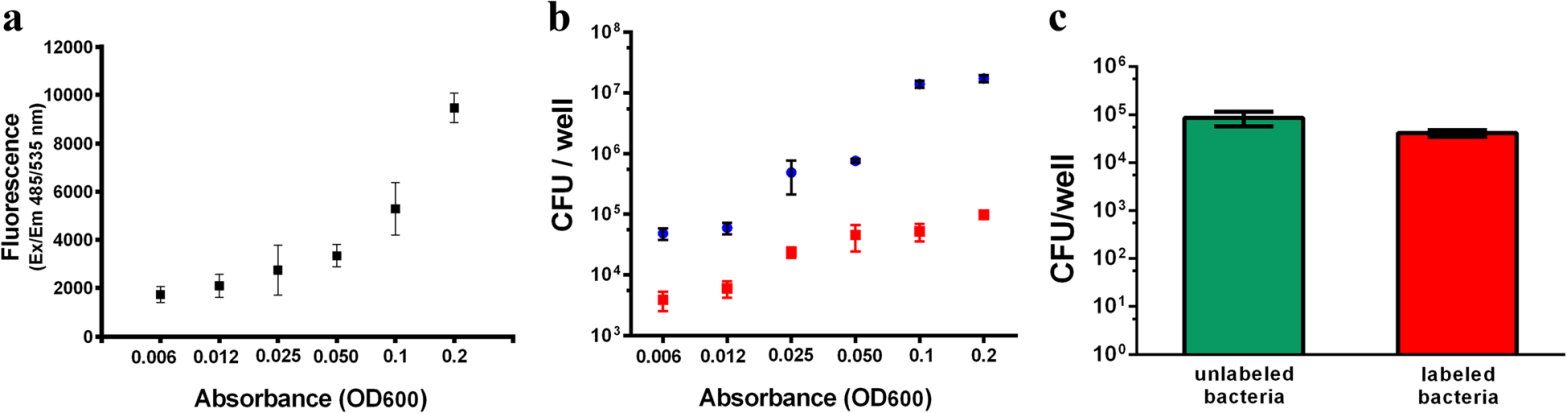
Adhesion of labeled *B. pertussis* Tohama I on A549 cells. **a** A549 cells were infected for 1 h with serially diluted labeled bacteria starting from an optical density (OD_600_) of 0.2. After washes, cell-associated bacteria were quantified by fluorescence reading at Ex/Em 485/535 nm. **b** After fluorescence reading, infected cells were treated with 1 % saponin, and lysates were serially diluted and plated for CFU counting of adhering bacteria (). Aliquots of serially diluted bacterial suspensions used for infection were also plated () **c** A549 cells were infected with unlabeled and labeled *B. pertussis* Tohama I at OD_600_ 0.2 for 1 h. Adhering bacteria were quantified by CFU counting as described before. Results represent mean ± SD from one out of the three representative experiments performed in triplicates

We set up a high-throughput *B. pertussis* adhesion assay to determine the level of adhesion of *B. pertussis* to the human alveolar epithelial cell line A549. We infected cells for 1 h with serially diluted *B. pertussis* Tohama I strain and after washes we measured the fluorescence of cell-adherent bacteria (Fig. 3a). Infected cells were subsequently treated with a detergent to release bound bacteria, and the number of cell-associated bacteria was determined by CFU counting and compared to the bacterial suspension at time zero (Fig. 3b). The results show that the measurement of bacteria-associated fluorescence was as sensitive as the conventional CFU counting in detecting the number of adherent bacteria.

Importantly, the presence of the fluorescent dye on the bacterial surface did not alter the adhesive properties of *B. pertussis* as demonstrated by comparing the level of adhesion of both labeled and unlabeled bacteria (Fig. 3c).

**Fig. 4:**
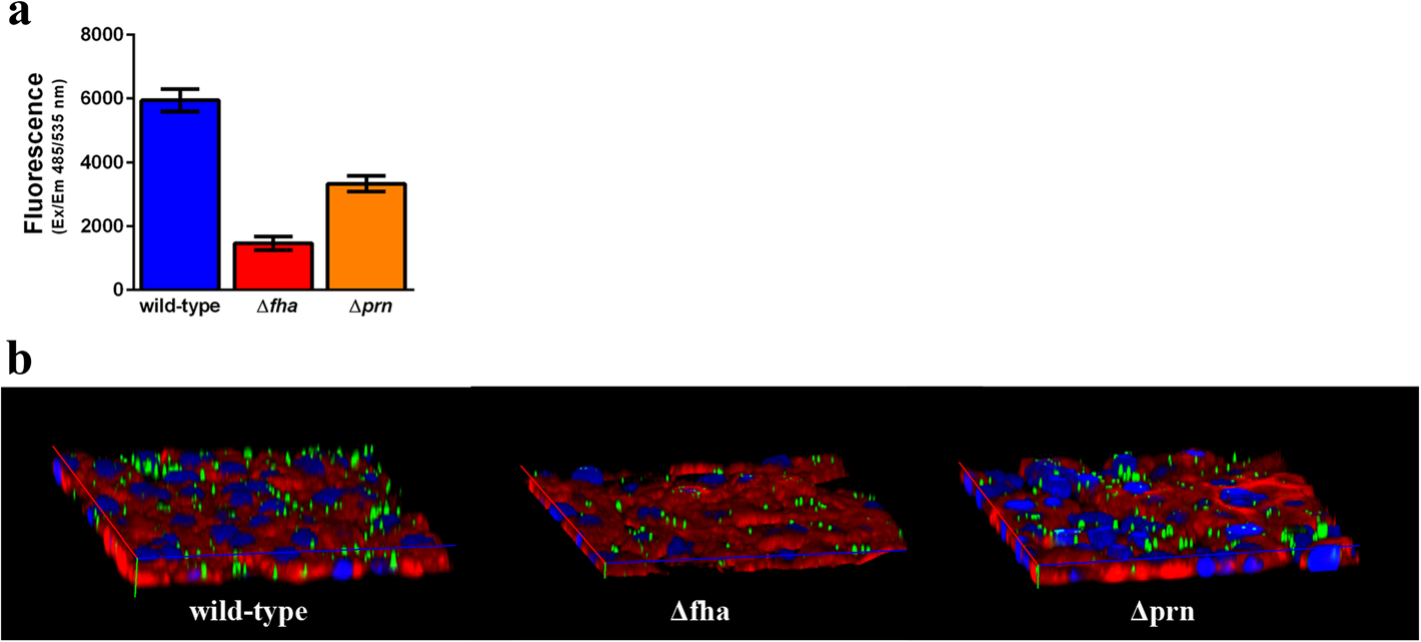
Contribution of FHA and PRN in *B. pertussis* adhesion to A549 cells. **a** A549 cells were infected with labeled *B. pertussis* strains BP536 (wild-type), BP102 (Δ*fha*) and BBC42 (Δ*prn*) at OD_600_ 0.1 for 1 h. After washes, cell-associated bacteria were quantified by fluorescence reading at Ex/Em 485/535 nm. Experiments were done in triplicates and data represent mean ± SD. **b** Adhesion assay was performed as described in A. Infected cells were fixed and actin (*phalloidin, red*) and nuclei (*DAPI, blue*) were stained for confocal microscopy analysis. Images are 3D view of Z-stack acquisition. Results represent mean ± SD from a representative experiment performed in triplicates

To evaluate the sensitivity of the assay in measuring the adhesiveness of bacteria to epithelial cells, we analyzed the contribution of the major adhesins of *B. pertussis*: filamentous hemagglutinin (FHA) and pertactin (PRN). We measured the adhesion of BP536-derivative mutant strains BP102 and BBC42, defective in FHA and PRN respectively. As expected these mutants were significantly less adhesive than the wild-type parental strain. The lack of FHA protein determined 75.2 % reduction in bacterial adhesion after 1 h infection on A549 cells, while the lack of PRN caused a 46 % decrease (Fig. 4a). These results were confirmed by confocal microscopy (Fig. 4b).

These data suggest that this assay represents a sensitive and automated quantification of adherent bacteria, and that it can be applied to compare the adhesive properties of different strains or evaluate the contribution of individual adhesins through the analysis of the relative knock-out strains.

**Fig. 5:**
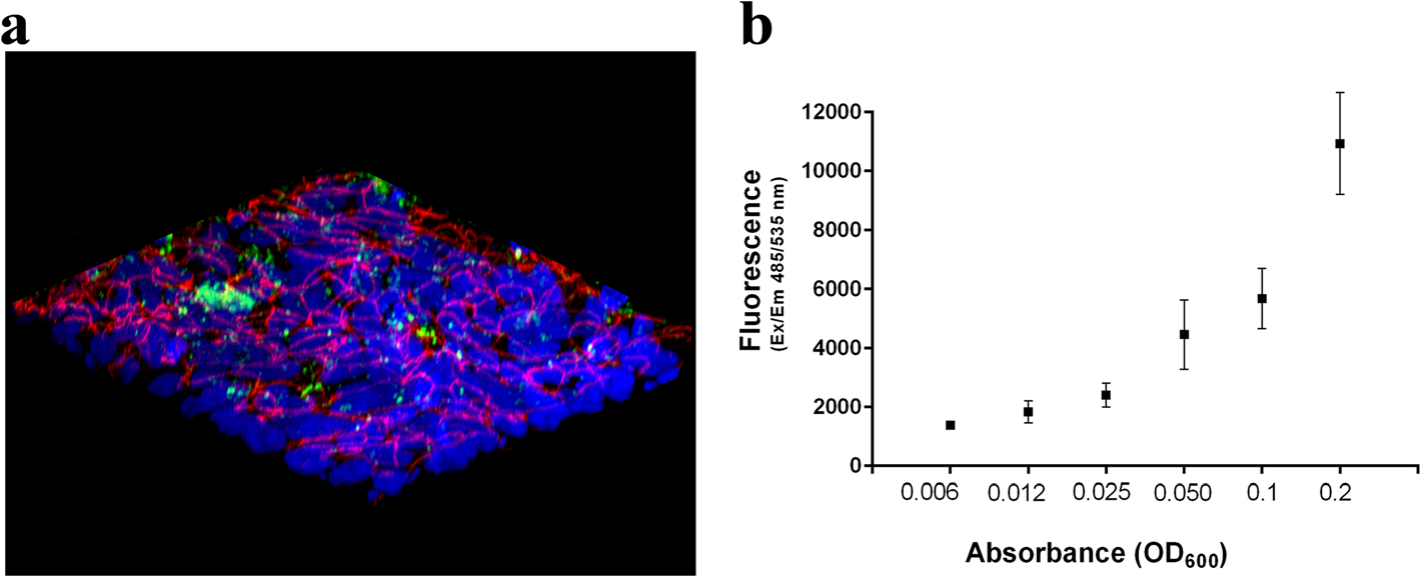
Adhesion of labeled *B. pertussis* Tohama I on polarized Calu-3 cells at air-liquid interface. **a** Polarized Calu-3 cells were infected with labeled *B. pertussis* Tohama I at OD_600_ 0.4 for 1 h. After extensive washes, infected cells were fixed and tight junctions (*ZO-1, red*) and nuclei (*DAPI, blue*) were stained for confocal microscopy analysis. Images are 3D view of Z-stack acquisition. **b** Polarized Calu-3 cells were infected for 1 h with serially diluted labeled bacteria starting from an OD_600_ of 0.4. After washes, cell-associated bacteria were quantified by fluorescence reading at Ex/Em 485/535 nm. Results represent mean ± SD from a representative experiment performed in triplicates

We further extended the method to a physiological 2D airway cell model by the use of Calu-3 cells grown on membrane supports at air-liquid interface. This cell model is characterized by distinctive columnar polarized cells and tight junctions (Fig. 5a), reproducing the features of functional human airway epithelium [16]. We infected polarized Calu-3 cells with *B. pertussis* Tohama I strain and verified the association of bacteria on the apical cell surface by confocal microscopy (Fig. 5a). After growing the cells on microplate membrane supports, we measured the level of adhesion by the previously described high-throughput method, determining the kinetic of association of fluorescent bacteria to this airway cell model (Fig. 5b).

### Inhibition of * B. pertussis* adhesion by vaccine-induced antibodies

A relevant application of this high-throughput adhesion assay is the evaluation of the ability of vaccine-induced antibodies to inhibit *B. pertussis* adhesion in vitro. Indeed, functional antibodies induced by vaccination may provide protection by several mechanisms, one of which is preventing bacterial adhesion to airway epithelial cells.

**Fig. 6:**
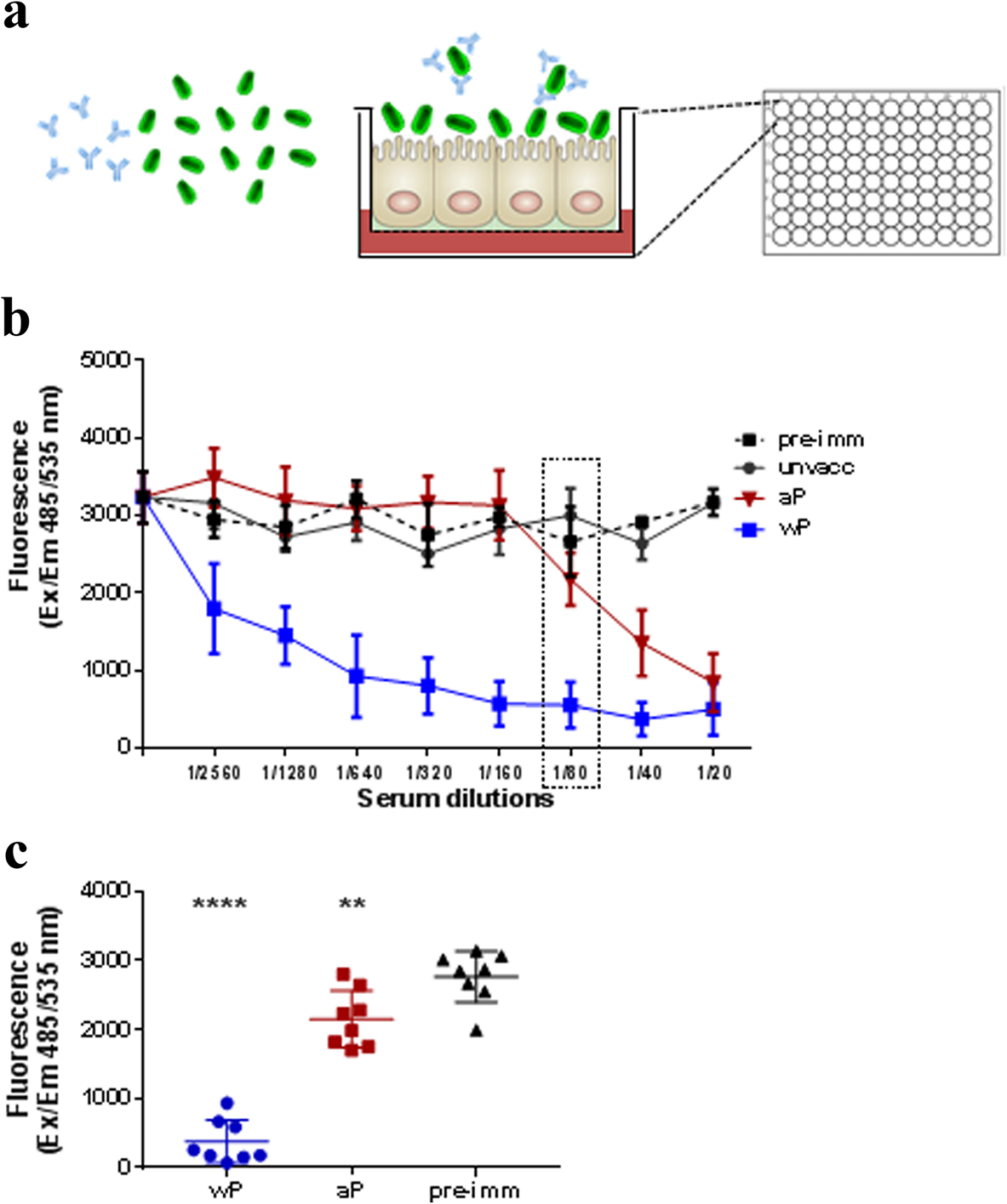
Impact of vaccine-induced antibodies on *B. pertussis* adhesion to A549 cells. **a** Schematic representation of the assay. **b** Sera from mice vaccinated with an acellular pertussis vaccine (aP) and a whole cell pertussis vaccine (wP) (8 mice/group) were pooled, serially diluted in infection medium and incubated with labeled wild-type *B. pertussis* Tohama I for 1 h. Pre-immune sera and sera from unvaccinated mice were used as negative control. A549 cells were then infected with the bacteria/sera mixtures for 1 h and, after extensive washes to remove unbound bacteria, cell-associated bacteria were quantified by fluorescence reading at Ex/Em 485/535 nm. Results represent mean ± SD from there independent experiments each performed in triplicates. **c** Sera from individual mice vaccinated with aP and wP vaccines (8 mice/group) were diluted 1 to 80 and adhesion assay was performed as described in B. Sera from unvaccinated individual mice were used as negative control. Results represent mean ± SD from a single experiment performed in triplicates; ****P < 0.0001, **P < 0.01

We considered in this study the ability of mouse antibodies induced by acellular (aP) and whole cell (wP) vaccines to inhibit adhesion of Tohama I strain to A549 cells. Fluorescent bacteria were pre-incubated with mouse pooled sera in a range of eight dilutions and A549 cells were infected as described before (Fig. 6a). We found that even though both aP and wP-induced antibodies interfere with bacterial attachment to epithelial cells, they showed different kinetics of inhibition (Fig. 6b): anti-wP serum showed a substantial reduction of adhering bacteria even at low serum concentrations, while anti-aP serum was effective in inhibiting only at the highest concentrations. Pre-immune serum and serum from unvaccinated animals were included in the experiments as negative control. We also measured the inhibitory effect of sera from individual mice (single serum dilution indicated in the dashed area of Fig. 6b), showing the consistency of the functional immune response in all animals vaccinated with aP and wP vaccines (Fig. 6c).

Overall, this assay allowed to determine the range of serum dilutions effective in inhibiting *B. pertussis* adhesion to epithelial cells and underlined the contribution of antigens included in aP vaccines in comparison to the entire membrane repertoire targeted by wP vaccines.

## Conclusions

This novel quantitative adhesion assay offers the possibility to analyze the functionality of vaccine-induced antibodies in inhibiting the bacterial adhesion to the host cells.

Considering the human-restricted host range of *B. pertussis*, the use of human cells resembling the airway mucosa is a valuable in vitro system to complement the studies in animal models. Moreover, in the perspective of limiting the use of animal testing, it is opportune to encourage and implement the development of methods in compliance with the 3Rs guidelines.

This assay is a sensitive, rapid and reproducible method to evaluate the capability of vaccine-induced antibodies to prevent bacterial adhesion and answer to the need of new pre-clinical tools to predict the impact of different vaccines on *B. pertussis* colonization.

## Methods

### Bacterial strains and growth conditions

The following *B. pertussis* strains were used in this study: Tohama I, BP536 [17], *fha*^*-*^ and *prn*^*-*^ derivative of BP536 (BP102 BBC42 in [18]). Bacteria were stored at −80 °C and recovered by plating on Bordet-Gengou (BG) agar plates, supplemented with 15 % sheep blood, for 3 days at 37 °C. Bacteria were then inoculated at initial 600 nm optical density (OD_600_) of 0.05–0.1 in Stainer-Scholte medium supplemented with 0.4 % (w/v) L-cysteine monohydrochloride, 0.1 % (w/v) FeSO_4_, 0.2 % (w/v) ascorbic acid, 0.04 % (w/v) nicotinic acid, 1 % (w/v) glutathione. Cultures were grown in rotary shakers at 37 °C for about 16 h.

### Sera

BALB/c mice (8 female/ group, 6-week-old) (Charles River Laboratories International Inc., Wilmington, MA) received two intramuscular immunizations, with a 4 weeks interval, with 100 μL (50 μL /leg) of Boostrix (GSK) and wP vaccine (NIBSC), both at one fifth of a human dose. Sera were collected before the vaccination and 2 weeks after the second immunization. Control unvaccinated mice were included in the experiments.

### Fluorescent labeling of bacteria

Alexa Fluor® 488 Carboxylic Acid, Succinimidyl Ester (Molecular Probes, Life Technologies) was dissolved in DMSO, aliquoted for single-use and stocked at −80 °C.

Bacteria were grown for 16 h in liquid culture. Bacteria were then pelleted at 8000 ×*g* for 5 min and resuspended in Dulbecco-PBS (D-PBS) at OD_600_ 0.5. A volume of 445 μL of bacterial suspension was then mixed with 50 μL NaHCO_3_ 1 M and 5 μL of Alexa Fluor® 488 and incubated for 15 min at 37 °C. After centrifugation at 8000 ×*g* for 5 min at room temperature, supernatant was removed and pellet was washed once with 1 mL PBS to remove unbound dye. Labeled bacteria were finally collected by centrifugation at 8000 ×*g* for 5 min and suspended in D-PBS at OD_600_ 0.2. Labeled bacteria were then 2-fold serially diluted in D-PBS and 100 μL of each bacterial dilution were transferred in triplicates in a black 96-well plate. Bacteria-associated fluorescence was assessed at 485/535 nm by a multi-well fluorescent reader.

The same bacterial suspensions were further serially diluted and plated on BG agar plates in triplicates for colony forming unit (CFU) counting.

### Cell cultures

Cells were grown at 37 °C in humidified atmosphere containing 5 % CO_2_.

A549 cell line (Human epithelial alveolar basal adenocarcinoma, ATCC CCL-185) was maintained in Ham’s F-12K medium (Life Technologies) supplemented with 10 % heat-inactivated fetal bovine serum (FBS, Gibco) and antibiotics.

Calu-3 cells (Human epithelial bronchial adenocarcinoma, ATCC HTB-55) were maintained in DMEM/F12 medium (Life Technologies) supplemented with 10 % heat-inactivated FBS and antibiotics.

In order to obtain a polarized epithelium, Calu-3 cells were cultured on HTS Transwell® 96-well permeable supports with 1 μm pore polyester membrane (Corning), previously coated with a 0.03 mg/mL rat tail collagen type I solution. Cells were seeded at density of 5x10^5^cells/cm2 (according to [16]) in the apical chamber and cell medium was added in the basal chamber. Once cells reached confluence, medium was removed and replaced only in the basal chamber, and cells were cultured at air-liquid interface (ALI) for the next 10–14 days. During ALI culture, basal medium was replaced every 2 days.

### High-throughput adhesion assay

A549 cells were seeded on black 96-well plate (2.5x10^4^/ well) and cultured for 1 day in absence of antibiotic. After fluorescent labeling, bacteria were suspended in F12-K medium OD_600_ 0.2. Bacteria were then serially diluted and 100 μL of each bacterial dilution was transferred in triplicates on plated A549 cells or on polarized Calu-3 cells. Infected cells were incubated for 1 h at 37 °C. After extensive washing to remove unbound bacteria, fluorescence was measured at excitation/emission 485/535 nm by Tecan Infinite F200PRO microplate reader.

To perform the serum-mediated BAI assay, pooled mouse sera were two-fold serially diluted in F-12K medium containing 1 % naïve mouse serum and pre-incubated with fluorescent bacteria for 1 h at 37 °C. To check the inhibitory activity of sera from individual mice, sera were analyzed at the single dilution of 1:80 and incubated with fluorescent bacteria as described before. The mixtures bacteria/sera were then added to cells; plates were centrifuged at 500 ×*g* for 5 min to synchronize the bacterial attachment and incubated for 1 h at 37 °C. After extensive washing, the cell-associated fluorescence was measured as described before.

In control experiments, after fluorescence reading, infected cells were lysed with 1 % saponin (Sigma) diluted in F-12K medium and solubilized bacteria were serially diluted and plated on BG agar plates for CFU counting.

### Immunofluorescence of fluorescent bacteria

After fluorescent labeling, bacteria were pelleted by centrifugation at 8000 ×*g* for 5 min and fixed in 3.7 % formaldehyde (Sigma) for 20 min. After multiple washes, bacteria were air dried on a poly-L-lysine coated glass slide. Samples were mounted with a drop of ProLong® Gold Antifade Mountant with DAPI (Molecular Probes, Life Technologies) and analyzed with Zeiss LSM710 confocal microscope.

### Qualitative analysis of bacterial adhesion by immunofluorescence

A549 cells were seeded on chamber slides coated with collagen I (5x10^4^/ well) and cultured for 1 day in absence of antibiotic, while Calu-3 cells were cultured at ALI for 10–14 days, as described previously.

A549 cells and ALI cultured Calu-3 cells were incubated with fluorescent bacteria as described in the previous paragraph. After washes, A549 cells were fixed with 3.7 % formaldehyde (Sigma) for 20 min and were then incubated with Alexa Fluor® 568 phalloidin (Molecular Probes, Life Technologies) diluted 1:400 in PBS + 0.1 % Tween20 + 1 % BSA for 30 min. Calu-3 cells were fixed directly on inserts by adding 3.7 % paraformaldehyde in the apical and basal chamber and were then permeabilized with 0.2 % Triton X-100 diluted in PBS for 5 min. Subsequently, samples were blocked with PBS containing 3 % Bovine Serum Albumin (BSA) (Sigma) for 15 min and incubated with rabbit ZO-1 polyclonal antibodies diluted in PBS + 1 % BSA (1:200) for 1 h. After washes, samples were incubated with Alexa Fluor® 568 goat anti-rabbit IgG (1:500) (Molecular Probes) for 30 min. After three washes with PBS, the insert was carefully detached by a scalpel and mounted on a microscope slide by adding one drop of ProLong® Gold Antifade Mountant with DAPI. Images were analyzed with Zeiss LSM710 confocal microscope.

## Abbreviations

ALI: Air-liquid interface
aP Vaccine: Acellular pertussis vaccine
BAI: Bacterial adhesion inhibition
BG: Bordet-Gengou
BSA: Bovine Serum Albumin
CFU: Colony forming unit
D-PBS: Dulbecco-phosphate buffered saline
FHA: Filamentous hemagglutinin
OD: Optical density
PRN: Pertactin
wP Vaccine: Whole cell pertussis vaccine
